# Waterborne cryptosporidiosis threat addressed.

**DOI:** 10.3201/eid0102.950211

**Published:** 1995

**Authors:** D G Colley


ommendations were developed with limited data and further research is needed to find cost-effective ways to control the spread of vancomycin resistance, HICPAC strongly encourages hospitals to develop their own institution-specific plans, which should stress the following elements: 1) prudent vancomycin use by clinicians, 2) education of hospital staff regarding vancomycin resistance, 3) early detection and prompt reporting of vancomycin resistance in enterococci and other gram-positive microorganisms by the hospital microbiology laboratory, and 4) immediate implementation of appropriate infection-control measures to prevent person-to-person transmission of VRE.

The recommendations were developed by HICPAC's Subcommittee on the Prevention and Control of Antimicrobial-Resistant Microorganisms in Hospitals and subject-matter experts and representatives of the American Hospital Association, American Society for Microbiology, Association for Professionals in Infection Control and Epidemiology, Infectious Diseases Society of America, Society for Healthcare Epidemiology of America, and Surgical Infection Society. The recommendations were published in February in *Infection Control and Hospital Epidemiology* 1995;16:105-13 and will also be published in the April 1995 issue of the *American Journal for Infection Control.*

**Hospital Infection Control**
**Practices Advisory Committee**
National Center for Infectious Diseases
Centers for Disease Control and Prevention
Atlanta, Georgia, USA

### Waterborne Cryptosporidiosis Threat Addressed

*Cryptosporidium parvum* was first recognized as a cause of human illness in 1976. From 1976 to 1982, the disease was reported rarely in the United States, primarily among the immunocompromised. In 1982, the number of reported cases began to increase dramatically along with the number of HIV-infected persons; outbreaks among immunocompetent populations also were reported. Recent municipal waterborne outbreaks of cryptosporidiosis in Texas (1984), Georgia (1987), and Oregon (1992), and a massive outbreak in Wisconsin in 1993 that affected more than 400,000 persons have raised awareness about the waterborne transmission of cryptosporidiosis. Since 1993, several smaller cryptosporidiosis outbreaks were reported in the United States: two were related to drinking water, six were linked to recreational water, and one was foodborne.

Cryptosporidiosis is caused by ingestion of the environmentally tough oocysts of the protozoan parasite *C. parvum*, an intracellular organism that can replicate in the gut epithelial cells of most mammals. Its oocyst is extremely resistant to chlorine, which is commonly used to treat municipal water.

In healthy persons, the disease lasts 1 to 2 weeks and can have considerable economic impact through absenteeism of those affected. In the immunocompromised, the disease is often severe, lifelong, and life-threatening. No effective therapy is available.

The magnitude of the 1993 Wisconsin outbreak and its association with a municipal water plant operating within existing state and federal regulations underlined the need for improved surveillance and coordination among public health agencies and spurred efforts for regulatory standards for *Cryptosporidium* in drinking water. During 1995-1996, the U.S. Environmental Protection Agency (EPA) intends to implement the Information Collection Rule, which requires utilities that serve populations of 100,000 or more and use surface water (lakes, rivers, streams) to test that water routinely for *Cryptosporidium* oocysts. If oocysts are found, the utility may also have to test finished water (tap water). Utilities that serve populations of 10,000 to 99,000 will also have to test source water, but for a shorter period. They will not be required to test tap water, even if oocysts are found. Authority to issue boil water advisories if oocysts are found varies from state to state. The health risks from ingesting low levels of *Cryptosporidium* are unknown. More than 300 representatives from 40 states and more than 25 regulatory, public health, water utility, and advocacy groups met at the Centers for Disease Control and Prevention (CDC) in Atlanta in September 1994 to discuss the prevention and control of waterborne cryptosporidiosis. Recommendations from the CDC workshop will be published in the next 2 to 3 months.

CDC held the first meeting of the Working Group on Waterborne Cryptosporidiosis in November 1994. The working group convenes biweekly by teleconference. For more information about the group, contact Margaret Hurd (phone: 404-488-7769, fax: 404-488-7761).

The working group has three main purposes: 1) promote a regular exchange of ideas, goals, activities, and proposals among individual scientists, agencies, and organizations interested in waterborne cryptosporidiosis; 2) make decisions on public health issues related to waterborne cryptosporidiosis; and 3) assemble smaller, more focused, task forces with expertise to develop, implement, and evaluate projects of the working group.

The working group has created task forces to assist local, state, and national public health departments, water utilities, and regulatory agencies in preparing for and managing outbreaks. The task forces have the following responsibilities:

- Develop and evaluate informational materials about cryptosporidiosis.
- Develop guidelines on when to initiate or end boil water advisories.
- Help formulate the language for EPA's Information Collection Rule.
- Identify officials with authority to issue boil water advisories. Examine legal issues associated with boil water advisories and the environmental testing, surveillance, and diagnostic requirements for waterborne *Cryptosporidium*.

In addition, technical task forces will collect data to develop guidelines for persons who may want to use bottled water and personal-use water filters and provide updates on the status of environmental sampling, water testing, and surrogate indicators of *Cryptosporidium* oocysts. These task forces will also report on the status of clinical diagnostic and serologic tools and provide local cryptosporidiosis infection rates to use in assessing the risk for waterborne transmission.

*C. parvum* oocysts are present in most surface water supplies; better technological tools and epidemiologic assessments are needed to determine the public health risks from these oocysts. Until the risks are fully known, efforts should be made to inform the public about cryptosporidiosis. Information on opportunistic infections, including cryptosporidiosis, for physicians who treat diseases in immunocompromised patients will be published this fall in a supplement to *Clinical Infectious Diseases* by authors from CDC and the Infectious Diseases Society of America.

**Daniel G. Colley**
National Center for Infectious Diseases
Centers for Disease Control and Prevention
Atlanta, Georgia, USA

